# STAIR CLIMB TIME AND FUNCTIONAL POWER ASSOCIATIONS IN EARLY OLD AGE: SWAN

**DOI:** 10.1093/geroni/igz038.1988

**Published:** 2019-11-08

**Authors:** Elsa Strotmeyer, Brittney S Lange-Maia, Jane A Cauley, Sheila A Dugan, Samar R El Khoudary, Kelley Pettee Gabriel, Nancy W Glynn, Carrie A Karvonen-Gutierrez

**Affiliations:** 1 University of Pittsburgh, Pittsburgh, Pennsylvania, United States; 2 Rush University Medical Center, Chicago, Illinois, United States; 3 University of Texas, Austin, Texas, United States; 4 University of Michigan, Ann Arbor, Michigan, United States

## Abstract

Stair climbing assesses neuromuscular components of movement, including muscle power (force*velocity) which may decline earlier in aging vs. strength. We hypothesized age and age-related factor (N=1370; 65.5±2.7 years) associations to stair climb total time (sec), ascend lap time degradation (lap 1 minus 3), power (W/kg body weight) and power degradation (lap 1 minus 3). Adjusting for demographic, lifestyle and age-related comorbidity factors using multivariate linear regression, older age independently related to slower total time and lower power. Non-white ethnicity had slower total time (Black, Hispanic), higher ascend time degradation (Hispanic), and lower power (Hispanic, Chinese, Japanese) vs. Whites. Higher 36-Item Short Form Health Survey (SF-36) and Modified Baecke physical activity scores indicated better performance: lower total time, higher power (SF-36 only), and less degradation in ascend time and power. Stair climb time and power in early old age may capture initial functional loss targets for interventions to prevent late-life disability.

